# Supplementary material for: Dating Skin Lesions of Forensic Interest by Immunohistochemistry and Immunofluorescence Techniques: A Scoping Literature Review
Source: Diagnostics (Basel). 2024 Jan 11;14(2):168. doi: 10.3390/diagnostics14020168 (PMC10814027; doi:10.3390/diagnostics14020168)
Supplement: Supplementary file 1 [file diagnostics-14-00168-s001.zip › diagnostics-2782716-supplementary.pdf]

Authors and date of publication	Study design	Markers	Techniques	Study objective	Sample size	Sample characteristics	Results	Statistical analysis
Zhong et Zhen (1991)[31]	Retrospective cohort study	Histamine (HA)	IF	Investigate microscopic HA quantification aimed at the timing of antemortem wounds.<br><br>Investigate the source of HA in the wound edges.	3	-Type of wound: Gunshot wounds: 2 cases<br>Skin abrasion: 1 case<br><br>- Wound age: unknown	-Mast cell count: no significant difference between injured skin and control samples ($p > 0.05$).<br><br>-HA content: Gradual increase up to 30 minutes after injury.<br><br>-Histamine fluorescence: Decrease observed in areas within 0-200 micrometers from the wound edge.	The results were statistically significant
Betz et al. (1992)[49]	Retrospective cohort study	α-actin positive myofibroblasts	IHC: avidin-biotinylated peroxidase complex (ABC) method and alkaline phosphatase and monoclonal antialkaline phosphatase (APAAP	Investigate the time-dependent appearance and disappearance of alpha smooth muscle actin in the granulation tissue in skin wounds	66<br>+<br>66 controls (uninjured skin samples)	Human skin wounds<br>-Type of wound: Human surgical wounds and lacerations<br><br>-Wound age: from 20 hours to 7 months<br><br>-Individuals age range: 15 to 92 years old	-Appearance of Alpha-SMA-positive myofibroblasts: typically, at around 5 days post-injury.<br><br>-Persistence in older wounds: observed in some cases with wounds aged over 2 months.	-

			complexes) method			-Time frame for data collection: autopsy conducted within 3 days after death.	-Estimation of wound age: presence of myofibroblasts can estimate a wound age of approximately 5 days or more.	
Betz et al. (1992)[51]	Retrospective cohort study	Collagen IV Laminin Heparan sulfate proteoglycan (HSPG)	IHC: ABC method	Investigate different markers for macrophage maturation to - contribute to the estimation of wound age	62 + 62 controls (uninjured skin samples)	Human skin wounds -Type of wound: surgical wounds, lacerations and stab wounds after surgical treatment<br><br>-Wound age: between 5h and 6 weeks	-Laminin and HSPG presence: wound age of at least 1.5 days.<br><br>-Pericellular collagen type IV presence: suggests a wound survival time of at least 4 days. Appears later than laminin or HSPG.	-
Betz et al. (1992)[75]	Retrospective cohort study	Fibronectin	IHC: ABC method	Investigate the time-dependent fibronectin reaction	53 + Not specified uninjured section stained without primary Ab	Human skin wounds -Type of wound: human surgical wounds, lacerations, stab wound, hematomas and abrasion<br><br>-Wound age: from few seconds to 6 weeks	In normal undamaged skin the endothelium basement membrane of larger blood vessels, epidermis and skin appendages showed a distinct, strong positive reaction.<br><br>In postmortem specimens, a granular	-

	- Individuals age range: 15 to 92 years old<br><br>-Time frame for data collection: autopsy conducted within 3 days after death.<br><br>Postmortem lesions caused by inguinal manipulations also tested.	or spot-like and sometimes a band-shaped positive reaction was observed focally which was exclusively restricted to the margins<br><br>Vital wounds - Group I (wound age from a few seconds up to 30 min):<br>10 out of 17 cases showed a clear and distinct positive fibronectin reaction. string-like ramifying and strongly reacting structures could be detected extending from the wound margin into the surrounding stroma<br><br>Vital wounds - group II (wound age more than 30 rain up to 3 weeks)<br>All specimens in this group showed enhanced positive reaction and especially a typical network of positive fibronectin strings containing numerous inflammatory cells	

							within the wound area<br><br>Vital wounds - group III (wound age ranging from 3 to 6 weeks)<br>a fibronectin pattern could be seen as previously described for the 2 cases in group II<br><br>No significant differences in the appearance of fibronectin depending on wound lo- calization, wound type or age of the individuals	
Betz et al. (1992)[48]	Retrospective cohort study	Collagen IV<br>Collagen VII	IHC: ABC method and APAAP method	Investigate on codistribution of both collagen IV and VII in the wound area	62<br>+<br>Unspecified control (uninjured skin samples)	Human skin wounds<br>-Type of wound: human surgical wounds, lacerations, stab wound after surgical treatment<br><br>-Wound age: from 1 day to 6 weeks<br><br>-Individuals age range: 15 to 92 years old<br><br>-Time frame for data collection: autopsy	-Appearance of basement membrane fragments: observed approximately 4 days after skin wound.<br><br>-Consistent presence of basement membrane fragments: in wounds over 13 days old.<br><br>-Complete basement membrane rearrangement:	-

						conducted within 3 days after death.	occurs at 8 days or more post-injury.<br><br>-Full basement membrane formation: typically observed at about 21 days or more after the wound.<br><br>-Distribution of collagens IV and VII: similar in wounds.	
Betz et al. (1993)[76]	Retrospective cohort study	Fibronectin<br>Collagen III<br>Laminin<br>Cytokeratin 5	IHC: ABC method	Investigate if these markers can provide useful information for an age-estimation of skin wounds obtained from putrified corpses.	20	Human skin wounds<br>- Type of wound: not specified<br><br>-Time frame for data collection: autopsy with postmortem interval between 3 days and 6 weeks with different grade of putrefaction	-It was observed that both laminin and cytokeratin 5 were better preserved compared to fibronectin and collagen type III.<br><br>-This suggests that laminin and cytokeratin 5 are more resistant to decomposition in the context of putrefied skin.<br><br>-In highly advanced putrefied skin, the detachment of epidermal layers was observed.	-

Betz et al. (1993)[77]	Retrospective cohort study	Ki67 antigen-positive cells	IHC: APAAP-method	Investigate if this protein can provide information useful for a wound age estimation	77<br>+<br>77 controls	Human skin wounds<br>-Type of wound: lacerations and stab wounds after surgical treatment.<br><br>-Wound age: from 3 hours to 7 months<br><br>-Individuals age range: 21 to 75 years old<br><br>-Time frame for data collection: autopsy conducted within 3 days after death.	-Ki67-Positive Cells: scarce in the central epidermis of reconstructed skin over wounds.<br><br>-Distribution: in 80% of the samples, Ki67-positive basal cells increased near the wound<br><br>- Fibroblastic cells: increase in positivity initially in wounds aged 1.5 days	-

Betz et al. (1993)[46]	Retrospective cohort study	Collagen type III<br>Collagen type V	IHC: ABC-method	Investigate on the location of interstitial collagen type V and compared these findings to the presence and position of collagen III.	79 (+ 15 wounds were produced postmortem in the same patients)<br>+<br>79 controls (uninjured skin samples)	Human skin wounds<br>-Type of wound: surgical wounds, stab wounds and lacerations after surgical treatment<br><br>-Wound age: from 8 h and 2.5 months	-For collagen III: not detected in wounds less than 2.5 days old. Became observable in wounds aged over 5 days with increased staining intensity as the wound aged.<br><br>-For collagen V: first observed in the lesioned area 3 days after the wound was inflicted.<br>Distinct positively reacting networks observed in wounds with shorter survival times were not present in the oldest wound.	-
Betz et al. (1993)[56]	Retrospective cohort study	Tenascin	IHC: ABC method	Investigate on localization of tenascin in human skin wounds	56<br>+<br>56 uninjured sections stained without primary Ab as controls	Human skin wounds<br>-Type of wound: surgical wounds, stab wounds and lacerations after surgical treatment<br><br>-Wound age: from 8 hours to 7 months<br><br>-Individuals age range: 15 to 92 years old died in traumatic events<br><br>-Time frame for data collection: autopsy	-Tenascin, appears around fibroblastic cells approximately 2 days after a skin wound.<br><br>-Network-like tenascin structures are first observed in wounds aged 3 days or more, becoming more regular in lesions aged 5 days or more.<br><br>-The staining intensity of tenascin	-

						conducted within 3 days after death.	decreases as the wound ages and can still be found in skin wounds around 1.5 months old.	
Betz et al. (1993)[77]	Retrospective cohort study	Collagen type I Collagen type VI	IHC: APAAP method	Evaluate the time dependent appearance of collagen types I and VI	74	Human skin wounds -Type of wound: surgical wounds, lacerations, and stab wounds after surgical treatment<br><br>-Wound age: from 1 day to 7 months	-Collagen I: positive reactions suggest a wound age of at least 5-6 days. Spot-like fragments around fibroblasts may appear in 4-day-old wounds.<br><br>-Collagen VI: positive reactions suggest a wound age of 3 days or more.<br><br>-Consistent Presence: both Collagen I and VI are consistently present after approximately 6-7 days post-injury.	-

Betz et al. (1994)[39]	Retrospective cohort study	Polymorphonuclear (PMN) cells<br>Fibroblastic cells<br>Epithelial cells<br>Non-Specific-Esterases<br>ATPase<br>Aminopeptidase<br>Acid phosphatase<br>Alkalin phosphatase	IHC	To investigate parameters useful for a forensic timing for human skin wounds	221<br>+<br>221 controls (uninjured skin samples)	Human skin wounds<br>-Type of wound: lacerations, surgical or stab/cut wounds<br><br>-Wound age: from few seconds to 7 months<br><br>-Individuals age range age: 15 to 94 years old<br><br>-Time frame for data collection: autopsy conducted within 4 days after death.	Wound Vitality Indicators<br>-Neutrophil Granulocytes: indicates immediate appearance after injury<br><br>Markers for Intermediate Time Frame<br>-Macrophages: presence indicates several hours to days since the injury<br><br>-Specific Particles: indicate several hours to days since the injury<br><br>Long-Term Wound Age Markers<br>-Hemosiderin Deposits: provide information on wound age over a longer period<br><br>-Pigments: provide information on wound age over a longer period<br><br>-Lymphocytic Infiltrates: provide information on wound age over a longer period	-

Betz et al. (1995)[78]	Retrospective cohort study	Markers for macrophage maturation:<br>27 E 10<br>RM 3/1<br>25 F 9<br>G 16/1	IHC	Investigate different markers for macrophage maturation to contribute to the estimation of wound age	117 vital skin wound<br>+<br>20 post-mortem wounds and skin specimens with beginning or advanced signs of putrefaction	Human skin wounds<br>-Type of wound: surgical wounds, lacerations and stab wounds<br><br>-Wound age: from few seconds to 7 months<br><br>-Individuals age range age: 16 to 83 years old	-Early Inflammation Marker (27 E 10): stained macrophages, monocytes, and neutrophilic granulocytes in normal skin and postmortem wounds.<br><br>-Intermediate Inflammation Marker (RM 3/1): specifically stained resident macrophages in postmortem wounds and undamaged skin. Increased number of migrated macrophages in wounds with a post-injury interval of 7 days or more, with positive results seen from 7 days to 7 months.<br><br>-Late Inflammation Marker (25F9): reacted positively with migrated macrophages and histiocytes. Positive results in skin wounds aged 11 days or more.<br><br>-Chronic Inflammation Marker (G 16/1): few	-

							histiocytes in normal skin.	
Hausmann et al. (1998)[79]	Retrospective cohort study	p53	IHC	Investigation on time-dependent expression of p53 protein during wound	82 + 20 controls (uninjured skin samples)	Human skin wounds -Type of wound: lacerations, stab wounds, surgical wounds.<br>-Wound age: from a few minutes to 11 weeks<br>-Individuals age range between 17 and 75 years	-Uninjured Skin: few p53-positive fibroblastic cells (r ≤ 0.1)<br>-Wounds Aged ≥ 3 Days: significant numbers of p53-positive fibroblastic cells (r ≥ 0.2)<br>-Post-Injury Interval: ratio > 0.5 suggests at least 8 days since injury	-

Dressler et al. (1998)[57]	Retrospective cohort study	Selectins	IHC: ABC method	Investigate on vitality and age of skin wounds by the detection of selectins.	197 (97 from autopsy material and 100 during surgical treatment)<br>+<br>31 post mortem skin injuries<br>+<br>97 controls (uninjured skin samples)	Human skin wounds<br>-Type of wound studied: lacerated/ contused wounds, incised wounds, surgical wounds and excoriations.<br><br>-Wound ages ranged: between 3 min and 790 days.	-P-Selectin: 3 minutes to 7 hours after injury<br><br>-E-Selectin: 1 hour to 17 days after injury. Significant decrease noted at 12 hours post-injury<br><br>-L-Selectin: detected on leukocytes in injured skin<br><br>-Leukocyte migration observed at 15-minute-old wounds	- The percentage of blood vessels with a positive reaction in injured and uninjured skin differed significantly ($P < 0.01$).<br><br>- E-selectin could be detected with low (+) to moderate (++) staining on endothelial cells in 51% of the cases, which was significantly higher ($P < 0.01$) compared with uninjured skin.<br><br>- A comparison of the distributions of the staining intensity in the skin wounds of the autopsy cases with those in the surgical cases revealed no significant difference for the P- and L-selectins ($P = 0.134$ and 0.824) but a significant difference for the E-selectin ($P = 0.017$)<br><br>- The comparison and distribution of immunohistological

staining reactions of the selectins of skin wounds induced postmortem with vital injuries (autopsies and surgical case material) showed significant differences ($P < 0.01$)

- A positive staining reaction for the E-selectin was found in skin wounds aged 1 h at the earliest and 17 days after injury at the latest; the intensity of the staining reaction was seen to decrease significantly 12 h after injury ($P < 0.05$)

Dressler et al. (1999)[33]	Retrospective cohort study	VCAM-1	IHC: ABC method	Investigate if there is a correlation between the occurrence of VCAM-1 and the wound age in injured skin	197 (97 from autopsy material and 100 during surgical treatment)<br>+<br>31 post mortem skin injuries<br>+<br>unspecified controls (uninjured skin samples)	Human skin wounds<br>-Type of wound: not specified<br><br>-Wound age: between 3 min and 790 days.	-VCAM-1 in Skin: Higher number of blood vessels exhibited a positive reaction compared to uninjured skin. Positive reactions observed on macrophages, cell detritus, perivascular inflammation cells, and a small percentage of keratinocytes.<br><br>-VCAM-1 in Postmortem-Induced Skin Wounds: most samples not showing VCAM-1 presence. Significant differences noted in the comparison and distribution of immunohistological staining reactions of VCAM-1 in postmortem-induced skin wounds compared to vital injuries.<br><br>-Time Dependence of VCAM-1 Expression: strong positive staining reactions observed as early as 3 hours and as late as	- The percentages of blood vessels with positive reaction in injured and uninjured skin differed significantly ($P < 0.01$)<br><br>- The comparison and distribution of immunohistological staining reactions of VCAM-1 of postmortem-induced skin wounds with vital injuries (autopsies and surgical case material) showed significant differences ($P < 0.01$).

							3.5 days in skin wounds. The intensity of VCAM-1 expression increased up to a wound age of 4-6 hours, followed by a subsequent decrease in staining intensity to the baseline level.	
Kondo et al. (1999)[21]	Retrospective cohort study	interleukin-1α (IL- 1α)	IHC: APAAP method	Investigate on temporal expression of interleukin-1α (IL-1α)	40 + 40 unwounded skin from same individuals as controls	Human skin wounds -Type of wound: stab wounds, incised wounds, surgical wounds and lacerations<br><br>-Wound age: from a few minutes to 21 days<br><br>-Individuals age range between 8 and 75 years old	-Early Wound Stages (Less than 30 Minutes):no infiltrating cells were observed in three wound specimens with wound ages of less than 30 minutes<br><br>-Appearance of Polymorphonuclear Neutrophils were first observed in a 4-hour-old wound.	Statistically, a significant difference in the mean ratio of IL-1α-positive cells was observed between wound specimens with wound ages ranging from 4 h to 1 day and those aged over 1.5 days ($P < 0.05$).

				- Time frame for data collection: autopsy conducted within 3 days after death.	-Neutrophils in Wounds (5 Hours to 1 Day): neutrophils continued to show a positive immunoreaction in wound specimens aged 5 hours to 1 day	

Grellner et al. (2002)[22]	Retrospective cohort study	IL-1β<br>IL-6<br>TNF-α	IHC: APAAP method	Investigate the correlation between Il-1, Il-6, TNF-α and age of skin damage	105<br>+<br>105 controls (uninjured skin)	Human skin wounds<br>-Type of wound: sharpe force from abdominal region, thoracic region, neck, dorsal region, extremities, head<br><br>-Wound age: from few seconds to 6 weeks<br><br>-Individuals age range between 3 and 93 years old<br><br>- Time frame for data collection: autopsy conducted within 4 hours and 8 days after death.	- IL-1β: the earliest observed reaction occurs at 15 minutes, while pronounced reaction manifesting between 30 to 60 minutes, and persistence up to 90 minutes.<br><br>-IL-6: the earliest observed reaction occurs at 20 minutes, with a pronounced reaction manifesting between 60 to 90 minutes, and persistence up to 5 hours.<br><br>-TNF-α: the earliest observed occurs at 15 minutes, with a pronounced reaction manifesting between 60 to 90 minutes, and persistence up to 3 hours.	-
Kondo et al. (2002)[23]	Retrospective cohort study	IL-8<br>MCP-1<br>MIP-1α	IHC	Investigate on the time-dependent expression of the chemokines	50<br>+<br>50 controls (uninjured skin sample)	Human skin wounds<br>-Type of wound: not specified	-Keratinocytes, sweat gland cells, and endothelial cells showed the presence of all three	- The positive ratios gradually decreased in groups III and IV. For IL-8, there were significant differences among

						-Wound age: from few minutes to 21 days<br><br>-Individuals age range between 7 to 77 years old<br><br>- Time frame for data collection: autopsy conducted within 3 days after death.<br><br>Group I 0-12 h<br>Group II 1-4 days<br>Group III 7-14 days<br>Group IV 17-21 days	chemokines in non-wounded specimens.<br><br>-Wound Age of 4-12 h: neutrophils were primarily located at the wound site. Some neutrophils contained IL-8, MCP-1, or MIP-1 a in their cytoplasm.<br><br>-Progression of Wound healing: macrophages started to dominate over neutrophils as the wound aged further.<br><br>-In Later Wound Stage:<br>IL-8, MCP-1, and MIP-1a were localized in the cytoplasm of macrophages and fibroblasts.	the four groups and in particular 15 out of 19 wounds in group II had IL-8 positive ratios > 50%.<br><br>- For MCP-1 and MIP-l(x significant differences were found between group I and the other three groups and be- tween groups II and IV. However, there were no significant differences between groups II and III and between groups III and IV
Kondo et al. (2002)[69]	Retrospective cohort study in humans	Ubiquitin	IHC	Investigate the time-dependent expression of ubiquitin	55<br>+<br>55 controls (uninjured skin samples)	Human skin wounds<br>-Type of wound: stab wounds, incised wounds, surgical wounds and lacerations<br><br>-Wound age: from few minutes to 21 days	-In unwounded skin nuclei of epidermal cells and sweat glands intensive Ub-positive reactions are present.<br><br>-Group I Early Wound: infiltration of neutrophils with strong intranuclear Ub-positive reactions	- In group II, the ratio of Ub-positive cells rapidly increased, and all wound specimens showed Ub-positive ratios of >10%<br><br>- The Ub-positive ratio gradually increased in group III, and all samples

	-Individuals age range between 8 to 75 years old<br><br>- Time frame for data collection: autopsy conducted within 3 days after death.	observed at wound sites.<br><br>-Group II Transition with Wound Age: mononuclear cells, likely macrophages, with intensive intranuclear positive reactions, were recruited at the wound sites.<br><br>-Group III Proliferative Phase (6 Days After Injury): new formation of granulation tissue exhibited intranuclear Ub-positive immunoreactions.<br><br>-Group IV Late-Stage Wound Healing: few leukocytes and fibroblasts showed faint Ub-positive reactions in the cytoplasm.	had a Ub-positive ratio of >20%<br><br>- A 7-day-old wound in group III showed the maximum value (45.3%) among all of the 55 human skin wound specimens in the present study.<br>The Ub-positive cell ratio in group IV in contrast, gradually decreased with increasing wound ages (mean±SE: 21.3±2.5%).<br><br>- Statistical analysis revealed that significant differences were found between group I and the three other groups, between groups II and III, and be-tween groups III and IV

Bonelli et al. (2003)[30]	Retrospective cohort study	Mast cells specific enzyme	IHC and Indirect IF	Investigate the time course of Mast Cells alterations in skin wounds	90<br>+<br>15 controls (uninjured skin samples)	Human skin wounds<br>-Type of wound: surgical wounds, lacerations and abrasions<br><br>-Wound age: from few second to 24 h<br><br>-Individuals age range: between 15 and 84 years old<br><br>- Time frame for data collection: 24h	- Mast cell count per unit area increases progressively with survival time, reaching a peak in subjects surviving 1-3 hours ($p<0.01$).<br><br>-After the 1-3 hour peak, mast cell count decreases.<br><br>-Mast cell count is lower than the control group if lesions occur more than 6 hours before death ($p<0.01$).<br><br>-Post-mortem lesions exhibit significantly fewer mast cells compared to other groups ($p<0.01$).	- Mast cell density appeared to vary significantly with time in vital lesions ($p<0.01$). This density increased progressively to a maximum in lesions which occurred 1–3 h before death and decreased thereafter, until in lesions which occurred earlier than 6 h it became lower than in controls.<br>- Mast cell density in post-mortem lesions was significantly less than in controls and in any other group of specimens

Hayashi et al. (2004)[36]	Retrospective cohort study	Vascular endothelial growth factor (VEFG)	IHC and IF	Investigate on time dependent expression of vascular endothelial growth factor (VEGF)	53 + 53 controls (uninjured skin samples)	Human skin wounds<br>-Type of wound: stab wounds, incised wounds, surgical wounds and lacerations<br><br>-Wound age: few minutes to 21 days<br><br>-Individuals age range: from 8 to 75 years old<br><br>- Time frame for data collection: autopsy conducted within 3 days after death.	-VEGF-positive signals detected in epidermal cells in unwounded skin.<br><br>-Wound specimens ages 4 hours to 1 day: neutrophils showed no immunopositive reaction for VEGF.<br><br>-VEGF localization: VEGF-positive signals localized in the cytoplasm of mononuclear cells and fibroblastic cells.<br><br>-Cell Types Producing VEGF: double-color immunofluorescence analysis revealed macrophages and myofibroblasts as the main cellular sources of VEGF in human skin wounds.	Statistical analysis demonstrated that significant differences were found between the four groups

Grellner et al. (2005)[34]	Retrospective cohort study	Transforming growth factor (TGF)-α TGF-β1	IHC: Labeled Streptavidin–Biotin (LSAB) method	Investigate on TGF-α and TGF-β1 for the determination of vitality and wound age	125 + 125 controls (uninjured skin samples)	Human skin wounds -Type of wound: sharp force from operation and autopsies<br><br>-Wound ages: few minutes 6 weeks<br><br>- Time frame for data collection: up to 8 days.	-TGF-α first reaction ca. 10 min; marked reaction in 30-60 min; persistence of reaction up to 6 days (in some cases);<br><br>-TGF-β1 first reaction in several minutes, marked reaction at 30-60 min; persistence of reaction up to 6 weeks	-
Bacci et al. (2006)[80]	Retrospective cohort study	Tumor necrosis factor-alpha (TNF-α) Avidin	IHC and IF	Investigation on the fraction of mast cells positive for TNF-a in skin lesions	50 + 10 controls (surgical biopsy of uninjured skin samples)	Human skin wounds - Type of wound: samples of vital skin lesions are surgical wounds, lacerations and abrasions<br><br>-Wound age: few seconds to 60 minutes.<br><br>- Individuals age range: from 15 to 86<br><br>- Time frame for data collection: 24-48h	-Progressive increase in mast cell numbers, becoming significant 1 hour after trauma.<br><br>-TNF-α expression in Mast Cells: the fraction of mast cells positive for TNF-alpha increased progressively after trauma, potentially playing an early role in directing tissue response to injury.	The differences among groups of biopsies were significant ($p<0.001$). In paired comparisons, a significant difference from controls was reached already in samples 16–30 min from lesions as well as in post-mortem lesions ($p<0.05$)

Ishida et al. (2008)[81]	Retrospective cohort study	Oxygen regulated protein 150 (ORP150)	IHC and Double-color IF	Investigate on ORP150 expression in human skin wounds of different wound ages for wound age determination.	58 + 58 controls (uninjured skin)	Human skin wounds<br>-Type of wound: stab wounds, incised wounds, surgical wounds, and lacerations<br><br>-Wound age: few minutes to 21 days<br><br>-Individuals age range: from 8 to 75 years old<br><br>- Time frame for data collection: autopsy conducted within 3 days after death.<br><br>Group I, 0–12 h<br>Group II, 1–5 days<br>Group III, 7–14 days<br>Group IV, 17–21 days	-Wound samples aged 4-12 Hours: neutrophils showed no ORP150-positive signals during this time frame.<br><br>-Wounds aged 7 days or more: macrophages and fibroblasts displayed ORP150-positive reactions during granulation tissue formation and angiogenesis.<br><br>-ORP150-Positive ratio:<br>the highest average ORP150-positive ratio was observed in group III (wound age 7-14 days).<br>All samples in group III had a ratio of >40%, with 17 samples showing a ratio of >50%.	Statistical analysis revealed significant differences between the first group and the other groups, between groups II and III, and between groups III and IV; however, there was no significant difference between groups II and IV
Ishida et al. (2009)[82]	Retrospective cohort study	CD45 Collagen type I	IF (double)	Investigate the time-dependent appearance of fibrocytes	53 + 53 controls (uninjured skin samples)	Human skin wounds<br>-Type of wound: stab wounds, incised wounds, surgical wounds, and lacerations<br><br>-Wound age: few hours to 21 days	-Wounds aged less than 3 days: CD45+/collagen type I+ fibrocytes were not detected in these wounds.<br><br>-Appearance of Fibrocytes: were first observed in wounds	Statistical analysis revealed significant differences between group III and the other three groups individually; however, there was no significant difference between groups II and IV

						-Individuals age range: from 8 to 75 years old<br><br>- Time frame for data collection: autopsy conducted within 3 days after death.<br><br>Group I, 0–3 days<br>Group II, 4–7 days<br>Group III, 9–14 days<br>Group IV, 17–21 days	aged 4 days. The presence of fibrocytes increased with wound age.<br><br>- The highest average number of fibrocytes was observed in group III wound (wound age 9–14 days).<br><br>- Presence of fibrocytes in human skin wounds indicates that the wounds are at least 4 days old	
Ishida et al. (2012)[44]	Retrospective cohort study	Cyclooxygenase-2 (COX-2)	IHC and double-color IF	Investigate on COX-2 expression in different wound ages for wound age determination.	60<br>+<br>60 controls (uninjured skin sample)	Human skin wounds<br>-Type of wound: stab wounds, incised wounds, surgical wounds, and laceration<br><br>-Wound age: few hours to 21 days<br><br>-Individuals age range: from 7 to 83 years old<br><br>- Time frame for data collection: autopsy conducted within 3 days after death.	-No significant leukocyte recruitment observed in unwounded skin and wounds less than 30 minutes<br><br>-Wound specimens aged 2 hours to 2 days: predominant presence of neutrophils labeled with myeloperoxidase (MPO) at the wound site. MPO-positive neutrophils expressed COX-2.	Statistical analysis revealed significant differences between group II and the three other groups, between groups I and III, and between groups III and IV; however, there was no significant difference between groups I and IV.

						Group I, 0–4 h<br>Group II, 8 h to 2 days<br>Group III, 3–9 days<br>Group IV, 12–21 days	-Wound specimens more than 3 Days: CD68-positive macrophages were recruited in addition to neutrophils and showed positive immunostaining for COX-2.	
Gauchotte et al. (2013)[19]	Retrospective cohort study	FVIIIra<br>CD15<br>Tryptase	IHC	Investigate the utility of three markers (FVIIIra, CD15, and tryptase) to interpret the timing of cutaneous stab wounds	70<br>+<br>32 controls (uninjured skin samples)	Human skin wounds<br>-Type of wound: stab wounds from autopsies and surgical specimens incision<br><br>-Wound age: Surgical incision in all cases to be <45 min.	-FVIIIra showed interstitial overexpression in all wounds but also in 53% of negative control wounds, resulting in high sensitivity (100%) but lower specificity (47%).<br><br>-CD15-positive cells were significantly higher at wound margins with a sensitivity (47%) and high specificity (100%). Sensitivity increased with time, reaching 50% after 20 minutes.<br><br>-Tryptase marker allow the assessment of vitality in 50 % of very recent wounds (<10 min).	- Interstitial overexpression of FVIIIra was found in 100 % (12/12) of medico-legal stab wounds, 100 % (58/58) of surgical wounds, and 53 % (17/32) of negative control wounds, leading to 100 % (95%CI = 94–100) sensitivity and 47 % (95%CI = 29–65) specificity.<br><br>- In medico-legal positive controls as well as in surgical wounds, the number of CD15-positive cells was significantly higher at wound margins than in the opposite control section (p = 0.0005 and p < 0.0001, respectively). CD15

				-CD15 and tryptase, but not FVIIIra, may be useful markers for differentiating recent antemortem from postmortem injuries.	positivity was significantly correlated with the time interval between incision and devascularization (p = 0.0005). No significant difference between the wound margins and the opposite section in the control wounds was observed (p = 0.14)<br><br>- Overall CD15 sensitivity was 47 % (95%CI = 33–60) and specificity was 100 % (95%CI = 87–100)<br><br>- No significant difference in the number of tryptase-positive cells was found between wound margins and opposite section in medico-legal wounds, surgical wounds, and controls. The degranulation rate was significantly higher in the wound margins of medico-legal stab

								wounds ($p = 0.03$) and surgical wounds ($p < 0.0001$). The degranulation rate was positively correlated with the time interval between incision and devascularization ($p = 0.0001$)<br><br>- A sensitivity of 60 % (95%CI = 47–73) and a specificity of 100 % (95%CI = 89–100) were established
Van De Goot et al. (2014)[18]	Retrospective cohort study	Fibronectin<br>CD62p<br>Factor VIII	IHC	To develop a wound age probability scoring system, based on the immunohistochemical expression levels of Fibronectin, CD62p and Factor VIII in wound hemorrhage.	424<br>+<br>383 controls (uninjured skin samples)	Human skin wounds from victims<br>-Type of wound: only wounds from blunt force trauma<br><br>-Wound age: from few seconds to 30 minutes<br><br>-Individuals age range: from 0 to 95 years old<br><br>- Time frame for data collection: autopsy conducted within 2 days after death	- For all three markers, in case of an IHC score 0, the probabilities that a wound was non-vital were highest. In case of an IHC score 1 or 2, the probabilities that a wound was a few minutes old were highest for all three markers. Finally in case of an IHC score 3, the probabilities that a wound was 15–30 min old were highest.	- For all three markers, in wounds of a few minutes old a significant increase ($p < 0.001$) in the IHC score was found compared with non-injured control skin samples<br><br>- The IHC scores for all three markers significantly increased even more ($p < 0.001$) in 15–30 min old wounds<br>-

Ishida et al. (2015)[74]	Retrospective cohort study in humans	Endotelial progenitor cells (EPCs)	Double- color IF	Investigate if EPCs would be useful for wound age determination	52 + 52 uninjured skin from the same humans as controls	Human skin wounds -Type of wound studied: stab wounds, incised wounds, surgical wounds, and lacerations<br><br>-Wound ages ranged: from few hours to 21 days<br><br>-Individuals age range: from 8 to 75 years old<br><br>- Time frame for data collection: autopsy conducted within 3 days after death	- CD34+ cells were observed in the unwounded human specimens, cells labeled with both anti-CD34 and anti-Flk-1, indicating EPCs, were not detected.<br><br>-EPCs were initially observed in human skin wounds with a post-infliction interval of 2 days.<br><br>-With the advancement of wound age, the number of EPCs increased progressively.	The results were statistically significant
Ishida et al. (2015)[42]	Retrospective cohort study	Matrix metalloprotein ase-2 (MMP-2) Matrix metalloprotein ases-9 (MMP-9)	IHC and double-color IF	Investigate on both MMP-2 and MMP-9 expression in human skin wounds for wound age determination.	55 + 55 controls (uninjured skin samples)	Human skin wounds -Type of wound: stab wounds, incised wounds, surgical wounds, and lacerations<br><br>-Wound ages: few minutes to 21 days	-Immunopositive reactions for MMP-2 were observed in all human skin specimens, including uninjured skin as a control.	Statistical analysis revealed that there were significant differences among group III and other three groups

						-Individuals age range: from 7 to 83 years old<br><br>- Time frame for data collection: autopsy conducted within 3 days after death<br><br>Group I (inflammatory phase), 0–3 days;<br>Group II (early proliferative phase), 4–7 days<br>Group III (late proliferative phase), 9–14 days<br>Group IV (maturation phase), 17–21 days	No MMP-9+ signals were detected in uninjured.<br><br>-The number of MMP-2+ macrophages significantly increased in accordance with wound ages.<br><br>- MMP-2+ cell number greater than 25 indicates a wound age of 9-12 days.<br><br>- MMP-9+ cell number exceeding 30 indicates a wound age of 3-14 days.	
Fronczek et al. (2015)[25]	Prospective cohort study in living human	MPO<br>CD45<br>CD68<br>MIP-1<br>IL-8<br>N(epsilon)-(carboxymethyl)lysine(CML)<br>Vitronectin	IHC	Investigate on inflammatory cells and inflammatory mediators in skin biopsies of wounds from living subjects to improve wound age determination.	101<br>+<br>Controls (unspecified)	Huma skin wounds from living patients<br>-Type of wound studied: bruises, abrasions, bites, stabs, scratches, unknown and firework.<br><br>-Wound ages ranged: from 4.5 hours to 25 days<br><br>-Individuals age range: 17–80 years	-Neutrophilic granulocytes: the number was highest in 0.2–2 days old wounds and gradually decreased over time.<br><br>-CD45-positive lymphocytes: the highest number was found in wounds of 0.2–2 days old, which decreased in wounds up to 10 days old.	- The number of neutrophilic granulocytes was significantly higher in wounds of 0.2–4 days old compared to wounds of 4–25 days old ($p = 0.004$). The same was true for wounds of 0.2–10 days old compared to wounds more than 10 days old ($p = 0.016$)

							-CD68-positive macrophages: the highest number was found in wounds of 2–4 days old.<br><br>- Inflammatory mediators: the highest number of inflammatory cells positive for MIP-1 were found in 0.2–2 days old wounds and decreased for older wounds.<br><br>-Vitronectin expression was only found in skin injuries up to 8 days old, with no significant differences between the wound age groups.	- No significant differences between the wound age groups were found ($p = 0.633$). The highest number of CD68-positive macrophages was found in wounds of 2–4 days old and then declined in time, with a significant difference between the wound age groups ($p = 0.014$)<br><br>- The number of macrophages in 0.2–4 days old wounds was significantly higher compared to 4–25 days old wounds ($p = 0.002$)<br><br>- Regarding MIP-1, IL-8, CML no significant differences were found between the wound age groups
Yagi et al. (2016)[26]	Retrospective cohort study and human skin wound specimens.	CD14<br>CD32B<br>CD68	IHC and double color IF	Investigate the time-course expression of CD32B and CD68 as well as CD14 to evaluate the effectiveness of	97<br>+<br>97 controls (uninjured skin samples)	Human skin wounds<br>-Type of wound: not specified<br><br>-Wound age: a few minutes to 30 days	-Immunohistochemical analysis indicated the presence of CD14-positive cells only on days 1-5 with sensitivity of 100%,	- The $CD14^{+}$ ratio in group 2 (100%; 50/50 samples) was significantly higher than in group 1 (7.9%; 3/38 samples) and group 3 (33.3%; 3/9 samples).

	combined assessment of wound age	-Individuals age range: from 16 days to 86 years old<br><br>- Time frame for data collection: autopsy conducted within 3 days after death<br><br>Group 1 (0–1 day) Group 2 (1–5 days), Group 3 (>7 days)	and specificity of 87.2%.<br><br>- Increase in CD14 expression on days 2-5 post-injury.<br><br>-The combination of CD14/CD32B/CD68 expression indicated a wound age of 1-5 days with high specificity.<br><br>The combination of CD14-/CD32B-/CD68-, suggested a wound age of less than 1 day.	$CD14^+$ represented wound ages of 1–5 days with a sensitivity of 100% and specificity of 87.2%<br><br>- The $CD32B^+$ ratio in group 2 (64.0%; 32/50 samples) was significantly higher than those in group 1 (28.9%; 11/38 samples) and group 3 (22.2%; 2/9 samples) and has a sensitivity of 64.0% and specificity of 72.3% in determining wound age in wounds 1–5 days postinfliction<br><br>- The $CD68^+$ ratios of group 2 (76.0%; 38/50 samples) and group 3 (88.9%; 8/9 samples) were significantly higher than that in group 1 (7.9%; 3/38 samples) and had sensitivity of 78.0% and specificity of 92.1% in identifying wound age in wounds of >1 day

Ishida et al. (2018)[64]	Retrospective cohort study	Aquaporin 1 (AQP1) Aquaporin 3 (AQP3)	IHC and double-color IF	Investigate the relation betweeen percentage of AQP1 & AQP3 and the wound age	55 + 55 controls (uninjured skin samples)	Human skin wounds -Type of wound: stab wounds, incised wounds, surgical wounds, and lacerations<br><br>-Wound age: a few minutes to 21 days<br><br>-Individuals age range: from 7 to 83 years old<br><br>- Time frame for data collection: autopsy conducted within 3 days after death<br><br>Group I (inflammatory phase), 0–3 days<br>Group II (early proliferative phase), 4–7 days<br>Group III (late proliferative phase), 9–14 days<br>Group IV (maturation phase), 17–21 days	-AQP3+ Keratinocytes: evident in groups II and III (4-14 days).<br><br>-AQP3+ cell number of >300 indicated wound ages of 5–10 days.<br><br>-AQP1+ markedly increased in group II.<br><br>-AQP1+ vessel area of over 5% implied wound ages of 4–12 days while positive area of >15% suggested wound age of 7–10 days.	Statistical analysis revealed that there were significant differences among group II and other groups.

Fouad et al. (2019)[27]	Retrospective cohort study	CD14 positive cells	IHC	Investigate on the efficacy of Cluster of Differentiation 14 (CD14) as reliable marker for estimating wound age	40<br>+<br>40 non-injured area as controls	Human skin wounds<br>-Type of wound studied: Stab wound, laceration,<br>Incised wound, Abrasion, Contusion<br><br>-Wound ages ranged: from few hours to 10 days<br><br>-Individuals age range: from 16 to 68 years old<br>- Time frame for data collection: postmortem interval was up to 3 days for all victims.	Expression of CD14<br>-Wounds Aged Less Than 12 Hours: staining percentage was 61.81±6.55%, primarily on polymorphs.<br><br>-Wounds Aged Between 12 and 24 Hours: gradual increase in CD14 expression (83.67±3.91%) on polymorphs and macrophages.<br><br>-Wounds Aged Between 1 and 3 Days: maximum CD14 expression (96.40±3.78%) observed with diffuse infiltration of macrophages and lymphocytes in the dermis.<br><br>-Wounds Aged More Than 3 Days: dramatic decrease in CD14 expression (14.80±3.49%) with few stained inflammatory cells and fibroblasts detected.	- There was a statistically significant relation be-tween percentage of CD14 expression and age of wound($p<0.001$).<br><br>- CD14 expression in group 3 (1-3days) was96.40±3.78%, which was significantly higher compared to groups 1, 2 and 4. On the other hand, CD14 expression ingroup 4 (>3 days) (14.80±3.49 %) was significantly lower compared to the other three groups

Kuninaka et al. (2020)[28]	Retrospective cohort study	CD11c^{+}HLA-DRα^{+} dendritic cells	Double color IF	Investigate on the relationship between DC number and wound age (analysis was carried out with anti-CD11c and -HLA-DRα antibodies)	53	Human skin wounds<br>-Type of wound: Stab wound, laceration, Incised wound and surgical wounds<br><br>-Wound age: from few hours to 21 days<br><br>- Time frame for data collection: autopsy conducted within 3 days after death	-No observation of CD11c+HLA-DRα+ DCs in uninjured skin samples and skin wounds aged 2 days or less<br><br>-Group I (Wound Ages 0-3 Days): limited or absent number of DC CD11c+HLA-DRα+.<br><br>-Group II (Wound Ages 4-7 Days): large number of CD11c+HLA-DRα+ DCs with all wound samples having more than 40 (mean ± SE: 57.7 ± 3.6).<br><br>-Group III (Wound Ages 9-14 Days): DC number of >40 (mean ± SE: 66.0 ± 2.9) in all samples.<br><br>-Group IV: significant decrease in the number of DCs (mean ± SE: 28.0 ± 2.5) compared with groups II and III	-No significant differences were observed based on age, gender, or wound type for the appearance of DCs<br><br>- The average number of DCs significantly decreased in group IV (mean ± SE: 28.0 ± 2.5), compared with groups II and III.

Jebur et al. (2020)[29]	Retrospective cohort study	Tryptase<br>IL-1β<br>IL-6	IHC	Evaluation of Mast cell tryptase as factor for wound vitality and IL-1 beta and IL-6 as parameters of wound age determination.	88 (are included samples and controls)	Human skin wounds<br>-Type of wound studied: Lacerated skin wounds classified into compression laceration, grinding laceration, cut laceration, tearing and crush injuries.<br>-Wound ages ranged: less than 12 hours<br><br>- Time frame for data collection: autopsy as soon as possible within a maximum time of 3 hours since arrival to mortality.	-Mast Cells Tryptase: higher infiltration of mast cells correlating with the passage of time in the study group.<br><br>- IL-1β Levels in Human Model: reverse relationship was found between the time of acute myocardial infarction (AMI) and the levels of IL-1 beta in the human model.<br><br>-IL-6 Levels in Human Model: The levels of IL-6 increased with the progression of AMI time.	The results were statistically significant

Bertozzi et al. (2021)[6]	Retrospective cohort study	CD15<br>CD45<br>IL-15<br>Tryptase<br>Glycophorin-A<br>MMPs	IHC	Investigate on the immunohistochemical behavior of samples collected from decomposed bodies (in different putrefaction phases).	7<br>+<br>1 control (uninjured skin sample)	Humans<br>-Type of wound studied: lacerated skin wound and incised skin wounds<br><br>- Time frame for data collection: different stages of decomposition between<br>a few hours and 15 days after death	-Most of the tested markers (tryptase, glycophorin, IL15, CD 15, CD 45, and MMP9) showed to be highly expressed in the tissue of putrefied skin for 15 days.<br><br>-MMP-2 was definitely positive in the only corpse with the most recent PMI.	-
Murase et al. (2022)[72]	Retrospective cohort study in humans	CHI3L1	IHC and fIHC	Investigate on some proteins of the C/CLP family could be used as markers of wound age estimation	187 samples (from 133 individuals)<br>+<br>not specified number of controls	Human skin wounds<br>-Type of wound: not specified<br><br>-Wound ages: from 0 to 13 days, subdivided in groups<br><br>-Individuals age range: from 0 to<br>95 years old<br><br>- Time frame for data collection: autopsy conducted within 3 days after death	In human cadaver skin wounds<br>-Days 0 to 1: weak CHI3L1 expression (0.11 ± 0.024)<br><br>-Days 2 to 3: Increased CHI3L1-positive cells (1.65 ± 0.19)<br><br>-Days 4 to 6: Further increase in CHI3L1-expressed cells (5.35 ± 0.35)<br><br>-Days 7 to 13: Decreased CHI3L1 expression (1.53 ± 0.24)	- The value of CHI3L1-positive cells of group III was significant compared with that of the other groups. Likewise, significant differences were observed in group II vs. group I and group IV vs. group I. There was no significant difference between group II and group IV<br><br>- Receiver operating characteristic (ROC) curve analysis indicated that

wounds from days 4 to 6 after injury could be clearly distinguished from other wounds.<br>Cutoff value: 2.75<br>Sensitivity: 92.31%<br>Specificity: 85.14%

Table S1 Table summarizing works related to studies on humans.

Authors and date of publication	Study design	Markers	Techniques	Study objective	Sample Size	Sample characteristics	Results	Statistical analysis

Zhong et Zhen (1991)[31]	Prospective cohort study	Histamine (HA)	IF: o-phthalaldehyde (OPT)	Investigate microscopic HA quantification aimed at the timing of antemortem wounds.<br><br>Investigate the source of HA in the wound edges.	86	Sprague-Dawley rats of either sex (weighing 100-150g)<br>- Type of wounds: Scissor wounds:<br><br>- Wound age: 5, 10, 15, 30, and 60 minutes before killing; postmortem wounds: 10, 30, and 60 minutes after death	-HA content: Gradual increase up to 30 minutes after injury.<br><br>-Histamine fluorescence intensity: Decrease observed in areas within 0-200 micrometers from the wound edge.	-Mast cell count: no significant difference between injured skin and control samples ($p > 0.05$).<br><br>-Fluorescence intensity and HA concentration: strong linear correlation with a regression coefficient of 0.97 ($p < 0.005$).

Kondo et al. (1996)[20]	Prospective cohort study	Il-1α<br>IL-1β<br>IL-6<br>TNFα	IHC: avidin-biotinylated peroxidase complex (ABC) + commercial enzyme-linked immunosorbent assay (ELISA) kits for the quantification of cytokines	Investigate the temporal local activity of inflammatory cytokines during the healing process of mouse skin wound, and the suitability of this procedure for wound age estimation	10<br>+<br>10 controls (uninjured murine skin samples)	Eight-week-old male Crj-CD1 (ICR) mice (weighing 30-35g)<br><br>-Type of wound: 1-cm-long Incised wounds made with a scalpel.<br><br>-Wound age: 0, 10 and 30 min, and 1, 3, 6, 12, 24, 72, 144 and 240 h.	1. Inflammation Stage:<br>- 3-6 hours: neutrophil infiltration positive for IL-10, IL-11, IL-6, and TNFα<br>- 24 hours: transition to IL-10, IL-11, IL-6, and TNFα-positive phagocytic macrophages<br><br>2. Cytokine Peaks:<br>- 3 hours: TNFα and IL-1β peak<br>- 6 hours: secondary smaller peak of IL-1β<br>- 12 hours: IL-6 peak<br><br>3. Proliferative Stage (72-240 hours):<br>- 72 hours: fibroblast migration and granulation tissue formation<br>- 240 hours: wound mostly healed<br><br>4. Cytokine "Rebound" Effect:<br>- All cytokines showed an increase at 72 hours post-wounding despite initial decreases.	The results were statistically significant
Grellner et al. (1998)[55]	Prospective cohort study	Fibronectin	IHC: alkaline phosphatase and monoclonal	Investigate on the expression of fibronectin in incised wounds in	6<br>+<br>6 controls (uninjured	6-months-old pigs (weighing 15-25kg)	- Positive reaction in 50% of cases	-

			antialkaline phosphatase (APAAP complexes)	porcine which were inflicted in the very early postmortem period.	murin skin samples)	- Type of wound: incised wounds measuring 1.5–2.0 cm in length and 1.0–1.5 cm in depth were set into the skin of chest, abdomen and hind legs<br><br>- Wound age: 5 min after circulatory arrest, sampled 12–14 h postmortem		
Kondo et al. (2002)[69]	Prospective cohort study	Ubiquitin (Ub)	IHC	Investigate the time-dependent expression of ubiquitin	Not specified	8-week-old Crj-CD1 (ICR) mice<br><br>Type of wounds: incised wounds measuring 1 -cm full-thickness made on the dorsal skin using a scalpel.<br><br>- Wound age: 12h, 1d, 3d, 6d, 10d	Strong intranuclear Ub-positive reactions were observed in 12 -h-old wound sites. At 6 days after injury, the proliferative phase of skin wound healing, and spindle-shaped fibroblastic cells in addition to mononuclear cells showed intranuclear Ub-positive immunoreactions	Morphometrical analysis demonstrated that the intranuclear Ub-positive ratio was most evident 6 days after injury.

Zhao et al. (2009)[66]	Prospective cohort study	p38MAPK<br>JNK<br>iNOS<br>eNOS<br>caspase-6<br>caspase-7<br>caspase-8<br>caspase-9<br>Calpain	IHC	Investigate the expressions and activities of calpain, p38MAPK, JNK, eNOS, iNOS, caspase-6, -7, -8, and -9 to explore the complex interaction for potential applicabilities as pameters to wound age estimation.	45<br>+<br>5 controls (uninjured murine skin samples)	Male 8- week-old adult healthy KM mice (weighing 30-35g)<br><br>-Type of wound: incised wounds 1.5 cm long full-thickness incision deep to the fascia was made with a scalpel<br>on the central dorsum<br><br>-Wound age: excision at 6 h, 12 h, 1 d, 3 d, 5 d, 7 d, 10 d and 14 d<br>post-incision.	Time-Dependent Protein Changes:<br>-p38MAPK peaked at 12 hours and 3 days post-injury<br>-p-JNK peaked at 1 day post-injury<br>-iNOS peaked at 1 day and 10 days<br>-eNOS, caspase-6, -7, -8, and -9 peaked at 3 days<br>-Calpain-1 peaked at 1 day and 5 days<br>- Calpain-2 peaked at 1 day and 5 days<br><br>Apoptotic Pathways: Activation of caspases-6, -7, -8, and -9 peaked at day 1	-<br>Morphometrically, the positive ratios of calpain-1 or -2-positive cells maximized at 1 d and 5 d post-wounding, then decreased gradually. The ratio of p38MAPKpositive cells maximized at 12 h and 3 d after wounding, and pJNK positive cells at 1 d and 7 d post-wounding. The ratio of iNOS-positive cells maximized at 24 h and 10 d, ratio of eNOS-po sitive cells reached its climax at 3 d after injury. Significant difference was found between the two groups of conterminous intervals<br><br>- The ratio of caspase-8-positive cells maximized at 1 d and 5 d post-wounding and ratios of caspase-9, -6, and -7-positive

cells reached its apex at 3 d post-wounding. The ratios decreased gradually at the following intervals. Significant difference was found between the adjacent two groups

Ma et al. (2011)[83]	Prospective cohort study	monoacylglycerol lipase (MGL)	Double direct and indirect IF	Investigate the expression of mono-acylglycerol lipase (MGL) during the skin-incised wound healing in mice and applicability of the time-dependent expression of MGL to wound age determination.	40 + 5 controls (uninjured murine skin samples)	Male adult healthy BALB/c mice (weighing 35–40g)<br><br>-Type of wound: 1.5-cm-long incision was made with a scalpel in the skin layer on the central dorsum<br><br>-Wound age: 6 h, 12 h, 1 day, 3 days, 5 days, 7 days, 10 days, and 14 days post-injury	-12 Hours Post-Injury: many polymorphonuclear cells (PMNs) at the injury site were MGL+/MPO+.<br><br>-3 Days Post-Injury: A significant number of mononuclear cells (MNCs) at the wound site were MGL+/F4/80+.<br><br>-7 Days Post-Injury: fewer double-positive cells were detected compared to earlier time points.<br><br>-1 Day Post-Injury: a few MGL+/α-SMA+ cells were present in the wound sites.<br><br>-After 10 Days Post-Injury: the number of MGL+/α-SMA+ cells gradually decreased compared to earlier time points.	Morphometrically, the average ratios of MGL-positive cells to total cells were over 50% at 5 and 7 days post-wounding, whereas it was <50% at the other posttraumatic intervals

Ishida et al. (2015)[74]	Prospective cohort study	CD34+/Flk-1+	IF - Fluorescence-Activated Cell Sorting (FACS)	Investigate the role of CD34+/Flk-1+ endothelial progenitor cells (EPC) in wound age determination	Unspecified + Unspecified controls (uninjured murine skin samples)	8- to 10-week-old male C57BL/6 mice<br><br>-Type of wound: dorsal skin generating six wounds using biopsy punch<br><br>-Wound age: of two and four days	Following the wound, the number of CD34+/Flk-1+ EPCs increased progressively, with a peak on day 4	Values at day 4<br>$P < 0.05$ vs. day 0

Barington et al. (2016)[24]	Prospective cohort study	MCA874G/MAC387 (MAC)	IHC	Estimate the age of bruises, focus has been on the changes over time not considering the force used to inflict the trauma by evaluating:<br><br>-Amount of hemorrhage<br><br>-Necrotic muscle fibers<br><br>-Infiltrations of neutrophils and macrophages	12<br>+<br>2 controls (uninjured porcine skin samples)	Female, Yorkshire-Landrace crossbred pigs (weighing 23–38 kg) –<br>Type of wound: blunt traumas were inflicted on the back along the M. longissimus dorsi of each pig during a period of 3-4 minutes using a mechanical device	-In dermis, MAC-positive neutrophils and macrophages were present after 2 hours, regardless of the force used.<br><br>-In the subcutis, infiltration of MAC-positive neutrophils and macrophages was observed after 2 hours<br><br>-The number of MAC-positive cells increased with both the age of the bruise and the force of impact.<br><br>-The severity of hemorrhage and the amount of necrotic muscle tissue were highly dependent on the force of impact.	-
Yagi et al. (2016)[26]	Prospective cohort study	CD14<br>CD32B<br>CD68	IHC<br>Double color IF	Investigate the time-course expression of CD32B and CD68 as well as CD14 to evaluate the effectiveness of combined assessment of wound age	34	6-week-old male BALB/c mice<br><br>-Type of wound: full-thickness wounds 4 mm in diameter were resected from the dorsum of each mouse using biopsy punch	- A significant increase in CD14 expression on days 2-5 post-injury compared to the initial time point and days 7 and 9. Immunohistochemical analysis indicated the presence of CD14-positive cells only on days 1-5. The	The results were statistically significant.

	-Wound age: 0 min, 1, 2, 3, 5, 7, and 9 days after wound infliction.	sensitivity for detecting wounds of 1-5 days using CD14 was 100%, with a specificity of 87.2%.<br>--The combination of CD14/CD32B/CD68 expression indicated a wound age of 1-5 days with high specificity. Another combination, CD14-/CD32B-/CD68-, suggested a wound age of less than 1 day.	

Wang et al. (2016)(59)	Prospective cohort study	IL-1b<br>IL-6<br>TNF-a,<br>IFN-g<br>MCP-1<br>CXCL12<br>VEGF-A<br>EGF<br>KGF<br>pro-col I α2<br>pro-col IIIα1	IHC (streptavidine-peroxidase method)<br><br>IF (triple)	Investigate on multiple biomarkers for wound age estimation.	54<br>+<br>6 controls (uninjured murine skin sample)	Male 8-week-old healthy BALB/c mice<br><br>-Type of wound: full-thickness dermal excisional wounds were created symmetrically over the midline on the dorsal skin by biopsy punch.<br><br>-Wound age: 12 h, 1 d,<br>3 d, 5 d, 7 d, 10 d, 13 d, 17 d and 21 d post-injury	-Neutrophils infiltrated the wound profoundly at 12 hours, while macrophages did so at 1 day and 3 days.<br><br>-Fibroblasts and fibrocytes accumulated in the wound from 3 days, with the highest transformation ratios observed at 7 days and 10 days, respectively (over 50%).<br><br>-MCP-1 and CXCL12 levels increased from 12 hours to 5 days, while IL-1b, TNF-a, and pro-col IIIa1 increased up to 7 days.<br><br>-IL-6 and VEGF-A showed an increase from 12 hours to 10 days.<br><br>-Pro-col Ia2 increased from 7 days to 21 days, while IFN-g decreased from 12 hours to 10 days.	- The average neutrophil number was over 50 per field at 12 h (102.6 ± 9.04, $p = 0.001$), and 1 d (80.6 ± 6.95, $p = 0.002$) post-injury<br><br>- The macrophage number as over 50 per field at 3 d<br>(64.8 ± 7.9, $p = 0.001$), 5 d<br>(92.8 ± 5.9, $p < 0.001$), 7 d<br>(80.0 ± 11.5, $p = 0.004$) and 10 d<br>(60.0 ± 8.9, $p = 0.004$) post-injury<br><br>- The fibroblast content increased from 3 d ($p = 0.035$), augmented at 5 d ($p = 0.001$) and peaked at 7 d ($p = 0.001$). It maintained at a high level up to 13 d ($p = 0.001$) and markedly decreased thereafter. Part of fibroblasts transformed into myofibroblasts from 5 d post-injury, with the highest

transformation ratio at 7 d ($p = 0.001$) to 10 d ($p < 0.001$) that over 50%

- Fibrocytes were occasionally detected at the bottom of the wound cavity at 3 d post-injury, then increased in number at 5 d ($p = 0.014$) and peaked at 7 d ($p = 0.005$). The fibrocyte-to-myofibroblast transformation was highest at 10 d ($p < 0.001$) with the ratio over 50%

- The expression of pro-col IIIα1 increased rapidly at 12 h and peaked at 1 d ($p < 0.001$), then decreased gradually in the following days and normalized at 10 d post-injury.

- The significant increase of pro-col Iα2 expression was observed from 7 d

								to 21 d, which was highest at 10 d ($p = 0.005$) to 13 d ($p = 0.005$) post-injury. IFN-γ showed a different expression pattern that decreased after injury, minimized at 5 d ($p < 0.001$) and resumed to the normal level at 13 d post-injury<br><br>- There were significant increases in the expressions of MCP-1, IL-1β and TNF-α from 12 h to 7 d post-injury, which peaked at 1 d ($p = 0.001$), 5 d ($p < 0.001$) and 7 d ($p < 0.001$) respectively. The CXCL12 expression was increased from 12 h to 5 d and peaked at 3 d ($p < 0.001$) post-injury
Abo El-Noor et al.(2017)(84)	Prospective cohort study	iNOS<br>IL-6	IHC	investigate the expression of iNOS and IL-6 proteins during skin burn injury healing for its forensic application in	50<br>+<br>Unspecified controls	Adult male albino rats (weighing 150 to 200 g)<br>-Type of wound studied: full-thickness (2cm x	-Ante-mortem Burn Healing: iNOS expression increased from days 1 to 7. Declined from days 9 until the end of the proliferative stage.	- ANOVA test showed statistically significant difference between the mean iNOS expression during the various studied

		determination of skin burn age and their possible role in differentiating between ante-mortem and post-mortem burn.		2cm) skin burns were made on rats with a heated soldering iron applied for 3 s.<br><br>-Wound age: sacrificed at 1, 3, 5, 7, 9, 11, 13, 15 and 21 days following the burn. Post-mortem burn was inflicted 6 h after scarification (5 rats)	iNOS expression observed in inflammatory cells, fibroblasts, and endothelial cells in the granulation tissue.<br>During the remodeling stage, iNOS expression mainly observed in macrophages.<br><br>-Post-mortem Burn Healing: iNOS protein expression declined significantly in all cells, with the lowest mean iNOS positive staining observed.<br><br>-IL-6 Expression: elevated in samples taken from the burn injury site throughout various time intervals studied. Expression was highest on day 5 and decreased gradually during the remodeling stage.	stages at the different time intervals<br><br>- Post hoc LSD test revealed significant differences between all the studied time intervals except day 1 versus day 11, Day 3 versus day 9, and day 5 versus day 9 (p values = 0.708,0.264, and 0.455 respectively)<br><br>- A statistically significant difference was found between the mean ranks of IL-6 expressions in the various studied stages at the different time intervals of burn healing.<br>- Post hoc test revealed non-significant differences between day 1 and day 11, day 3 and day 9, day 5 and day 7, day 11 and day 13 (p values = 0.067, 0.875, 0.806, and 0.671 respectively)

Spearman's rank correlation between iNOS and IL-6 expressions during the studied stages of burn healing revealed a statistically significant positive association (r = 0.74, p value < 0.001) between the two markers. Both increased gradually in inflammatory and early proliferation stages and started to decrease gradually in late proliferative stage and remodeling stage while reaching the minimum at the postmortem stage.

Khalaf et al. (2019)(37)	Prospective cohort study	Macrophage specific gene CD68<br><br>Alpha-smooth muscle actin (α-SMA)<br><br>Vascular endothelial growth factor (VEGF)<br><br>TGFb1	IHC	Investigate on the potential capacity of CD68, α-SMA, VEGF, and TGFb1 to be used as biomarkers for wound age determination.	18<br>+<br>Unspecified controls	Male Wister rats (weighing 170-200 g)<br><br>Rats skin wounds<br>-Type of wound: Full-thickness (extending up to adipose tissue) circular wounds (2 cm^2 × 2 cm^2) on dorsal back of the animal was created using a sterile biopsy punch.<br><br>-Wound age: 0, 1, 3, 5, 7, and 14 days post-injury.	-Days 0, 1, and 3: skin tissue showed negative to mild α-SMA and VEGF expression.<br><br>-Day 3: high infiltration of CD68+ macrophages, but negative at days 0 and 1.<br><br>-Days 5-7: all markers (α-SMA, VEGF, CD68) strongly expressed, peaking at day 7.<br><br>-Day 14: except for α-SMA markers not expressed.	A significant upregulation in both VEGF and TGFb1 mRNA levels was observed in all of the vital skin wounds at the early stages (0, 1, 3, and 5 days) and reached to the peak at day 7. However, a sharp downregulation was detected in the mRNA level of both the VEGF and TGFb1 at day 14
Murase et al. (2022)(72)	Prospective cohort study	CHI3L1 (protein and mRNA)	IHC<br>IF	Investigate on some proteins of the C/CLP family could be used as markers of wound age estimation	48<br>+<br>8 mice were euthanized immediately after injury and acted as controls	6-week-old male BALB/cCrSlc mice<br><br>-Type of wound: six full-thickness excisional wounds were applied on the dorsal skin using a 4-mm biopsy punch<br><br>-Wound ages ranged: 1, 2, 3, 5,	CHI3L1 Expression In murine skin wounds, the expression of CHI3L1 changed over time.<br><br>Receiver operating characteristic (ROC) curve analysis indicated that wounds from days 4 to 6 after injury could be clearly distinguished from other wounds. Cutoff value: 2.75	- Chi3l1 rapidly strengthened to nearly 28.5 times in the day 1 group compared with that in the control group and dropped from days 2 to 9. There were significant differences between days 1, 2, 3, 5, and 7 and the control group<br><br>- CHI3L1 expression

			7, and 9 days after injury	Sensitivity: 92.31%<br>Specificity: 85.14%	increased from day 1 and showed a peak increase by approximately 54.9 times in the day 2 group compared with the control. The protein levels then gradually decreased until day 9. Statistically significant differences were found between days 1, 2, 3, and 5 and the control

Table S2 Table summarizing works related to studies on animals.
